# Identifying “problematic” clusters in record linkage using graph measures

**DOI:** 10.23889/ijpds.v9i5.2560

**Published:** 2024-09-18

**Authors:** Tony Stone

**Affiliations:** 1 University College London

Manual review of outputs from record linkage processes remains a frequently necessary but time-consuming operation. This work outlines a potential means to reduce the manual review workload through the extraction of graph measures from the outputs of record linkage processes combined with machine learning classification methods.

The outputs of a pairwise record linkage process can naturally be represented by undirected simple graphs with a vertex representing each record and an edge representing each comparison of records. One representation may be a graph which only includes edges representing pairwise comparisons that have a match score greater than a given threshold. A second representation may include match scores (suitably transformed) as the weight of the edges (pairwise record comparisons) connecting vertices (records). A third representation may include not just the overall match score as a weight on each of the edges (pairwise record comparisons) but also, for each compared attribute (e.g. names, date of birth, address, etc.), the contributing component to the overall match score as additional edge attributes.

In this work, a "problematic" cluster is a set formed of three or more records which, in totality, belong to two or more true entities. Equivalently, a connected graph of three or more vertices (representing records) with at least one edge that represents a false positive link.

Finally, this work evaluates the performance and generalisability of various combinations of graph measures for identifying problematic clusters across linkage processes featuring varying degrees of corruption and duplication.

